# Host Cell Targets for Unconventional Antivirals against RNA Viruses

**DOI:** 10.3390/v15030776

**Published:** 2023-03-17

**Authors:** Vicky C. Roa-Linares, Manuela Escudero-Flórez, Miguel Vicente-Manzanares, Juan C. Gallego-Gómez

**Affiliations:** 1 Molecular and Translation Medicine Group, University of Antioquia, Medellin 050010, Colombia; 2 Molecular Mechanisms Program, Centro de Investigación del Cáncer, Instituto de Biología Molecular y Celular del Cáncer, Consejo Superior de Investigaciones Científicas (CSIC), University of Salamanca, 37007 Salamanca, Spain; miguel.vicente@csic.es

**Keywords:** host-targeted antivirals, RNA viruses, drug repositioning

## Abstract

The recent COVID-19 crisis has highlighted the importance of RNA-based viruses. The most prominent members of this group are SARS-CoV-2 (coronavirus), HIV (human immunodeficiency virus), EBOV (Ebola virus), DENV (dengue virus), HCV (hepatitis C virus), ZIKV (Zika virus), CHIKV (chikungunya virus), and influenza A virus. With the exception of retroviruses which produce reverse transcriptase, the majority of RNA viruses encode RNA-dependent RNA polymerases which do not include molecular proofreading tools, underlying the high mutation capacity of these viruses as they multiply in the host cells. Together with their ability to manipulate the immune system of the host in different ways, their high mutation frequency poses a challenge to develop effective and durable vaccination and/or treatments. Consequently, the use of antiviral targeting agents, while an important part of the therapeutic strategy against infection, may lead to the selection of drug-resistant variants. The crucial role of the host cell replicative and processing machinery is essential for the replicative cycle of the viruses and has driven attention to the potential use of drugs directed to the host machinery as therapeutic alternatives to treat viral infections. In this review, we discuss small molecules with antiviral effects that target cellular factors in different steps of the infectious cycle of many RNA viruses. We emphasize the repurposing of FDA-approved drugs with broad-spectrum antiviral activity. Finally, we postulate that the ferruginol analog (18-(phthalimide-2-yl) ferruginol) is a potential host-targeted antiviral.

## 1. Introduction

Viral diseases are an important focus of study in biomedical sciences due to their impact on human health. Many RNA and DNA viruses infect vertebrates, including humans. RNA viruses have a notable impact as they cause severe diseases with an important socio-economic burden, e.g., acquired human immunodeficiency (HIV), hemorrhagic diseases (Ebola, Zika, DENV), hepatitis C (HCV), or influenza [1]. Human intervention in natural ecosystems and the continuous growth of the human population have led to increased human contact with zoonotic virus reservoirs and an increased frequency of interspecies infection, as illustrated by the COVID-19 pandemic, caused by coronavirus SARS-CoV-2 [2].

Although antiviral drugs and vaccines for human RNA viruses do exist, a major challenge is the emergence of drug-resistant variants [3]. This phenomenon is due to several factors, particularly that RNA produced by RNA virus-encoded RNA polymerases have a very high mutation rate (one per each 100,000 nucleotides) [4]. In addition, although reverse transcriptase has a lower probability to induce mutations (five in the full genome) [5], together both phenomena produce a very high rate of spontaneous mutation compared to the rate of mutation in, for example, eukaryotic cells [6]. In most cases, these mutations put the mutated virus at an evolutionary disadvantage by, for example, decreasing its rate of replication or impairing the proper assembly of the capsid. However, random mutation sometimes leads to the selection of antiviral-resistant variants (quasispecies) in which viral replication and/or infective capability remain unaffected or even improve [7]. Coronaviruses (CoVs) display a lower rate of random mutation, as they express nonstructural protein (nsp) 14. nsp14 is a 3′-5′ exoribonuclease with proofreading activity [8]. The rapid emergence of SARS-CoV-2 variants (e.g., the omicron variants and its subvariants) insensitive to vaccines suggests that this mechanism is not as efficient as originally proposed. Another compensating factor is the infectivity and replicative speed of SARS-CoV-2. By jumping quickly from cell to cell and propagating to multiple individuals in a population, the rate of mutation is elevated despite the existence of proofreading mechanisms [9]. Dengue, Zika, and Chikungunya have no currently approved treatments or effective prevention alternatives.

At present, antiviral drug development has focused on the identification of viral proteins and/or structures as potential targets for different compounds and small molecules, termed direct-acting antivirals (DAAs) [10]. DAAs are useful against DNA viruses due to the comparatively low rate of spontaneous mutations [11]. However, their efficacy against RNA viruses is less consistent [12], and each DAA needs to be evaluated independently. Nevertheless, there are DAAs with documented efficacy against RNA viruses, e.g., transcriptase reverse inhibitors against HIV (reviewed in [13]) or remdesivir, which targets the RNA polymerase of SARS-CoV-2 [14].

An alternative approach is to identify antivirals that do not select drug-resistant strains and are directed against the host factors required for productive viral infection. Host-targeted antivirals (HTAs) could be more effective against RNA viruses because host factors are genetically more stable than viral factors, potentially overcoming viral heterogeneity and the emergence of drug-resistant mutants [15]. Additionally, exploring FDA-approved drugs with HTA properties (drug repositioning) reduces time and optimizes economic resources [16]. For example, antitumor small molecules are an attractive option due to the convergence of some signaling pathways involved in cancer and infection caused by RNA viruses [17].

Here, we review recent evidence positioning several HTAs for the treatment of human diseases caused by RNA viruses. As a proof of principle, we focus on the case of an abietane ferruginol analog endowed with a broad antiviral spectrum against dengue, Zika, and herpesvirus [18,19]. These approaches suggest that repurposing current HTAs is a viable strategy that may be useful for the treatment of RNA viral infections [20]. We illustrate this point by describing the cases of several antitumor drugs that have displayed promising results against SARS-CoV-2 infection [20,21,22] as well as other RNA viruses [23,24] by targeting host cell mechanisms.

## 2. Cellular Factors Used by RNA Viruses in Their Replicative Cycle

Viruses are obligate intracellular parasites that require infection of living organisms (cells) to replicate [25]. In the course of their replicative cycle, viruses can modulate a wide variety of cellular processes that encompass the remodeling of the endomembrane system [26], cytoskeletal polymerization and organization [27] dynamics [19], modulation of gene and host protein expression [28], apoptosis and autophagy [29,30], cell division [31], evasion of the immune response [32], induction of epithelial to mesenchymal transition (EMT) [17], and regulation of lipid metabolism [33].

Viruses use diverse cellular receptors to attach and enter the host cell. Heparan sulfate receptors and other sulphated glycans are widely used by several families of viruses, including DENV [34] and some alphaviruses [35]. DENV, HCV, HIV, and Ebola viruses trigger clathrin-dependent receptor endocytosis [36,37,38,39]. Influenza and Ebola viruses also enter cells through pinocytosis [39,40], and SARS-CoV-2 infects cells via pH- and receptor-dependent/lysosomal entry [41]. After virion internalization and the release of the viral genome into the cytoplasm, positive-strand RNA viruses modify the cellular endomembrane system [42,43,44], forming structures known as “viral factories” [45].

The flavivirus genus produces at least two types of intracellular membrane structures: vesicle packets (VPs) and convoluted membranes (CMs). Replication of the viral genome happens at VPs, whereas RNA translation/polyprotein processing occurs at CMs [42,43]. CMs are formed through the membrane remodeling of the endoplasmic reticulum, whereas VPs seem to be derived from the Golgi apparatus [46]. The development of these structures is induced by changes in the lipid composition, the influence of integral membrane proteins, the activity of diverse cytoskeletal proteins and microtubule motors, and scaffolding by peripheral and integral membrane proteins [47]. Additionally, the endoplasmic reticulum and Golgi apparatus provide viruses with different sets of host proteins required for the processing, folding, and function of viral glycoproteins. Examples include ER α-glucosidases [48] and other cellular proteins that cleave viral proteins into their mature/active forms, such as furins [49].

The cytoskeleton is also heavily involved in the development of the viral cycle [50,51]. For example, Ebola virus hijacks microfilaments to transport viral nucleocapsids from viral replication centers to membrane budding sites [52], similar to vaccinia virus [53]. Actin filaments are also crucial during DENV internalization and the release of new virions [54]. Many viruses, e.g., influenza, HIV, and Ebola, co-opt microtubule-associated molecular motors for intracellular motility [55]. Influenza virus uses microtubules to move ribonucleoproteins from the nucleus to plasma membranes [56]. HIV uses microtubules to facilitate group-specific antigen (gag) trafficking and virus particle production [57,58], while Ebola virions hijack microtubules within membranous compartments to travel to the acidified vesicular compartment, where viral and cellular membrane fusion occurs [59].

RNA viruses not only hijack pre-existing cellular machinery to propagate infection but also trigger cellular modifications that favor viral dissemination. One crucial example is the virus-induced epithelial-to-mesenchymal transition (EMT).

The EMT is a trans-differentiation process that has been classically associated to development and the metastasis cascade [60]. It is an epigenetic program characterized by a progressive loss of epithelial polarity, the acquisition of individual cellular motility, and an invasive capacity [61]. During this process, epithelial cells adopt a fibroblastic, mesenchymal-like morphology. Viral triggers of the EMT include the AKT-mediated phosphorylation of β-catenin (HCV), which triggers its translocation to the nucleus and the induction of mesenchymal genes as well as the repression of E-cadherin transcription [62]. Recent data demonstrated that DENV infection activates the PI3K/Akt/Rho GTPase pathway, resulting in the activation of Rac1 and Cdc42 Rho GTPases, which in turn induce a lamellipodia and filopodia extension [63]. These cytoskeletal rearrangements highlight the convergence of virus-induced actin remodeling with an EMT induction.

**Figure 1:**
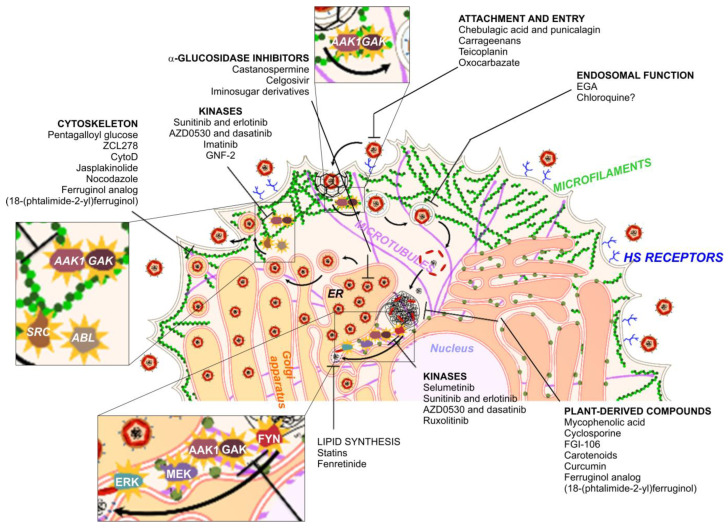
Host factors required for RNA virus entry and propagation. Host-targeted antivirals are indicated in the figure according to their presumed point of interference with the viral infectious cycle. ER: endoplasmic reticulum; EGA: bromobenzaldehyde N-(2,6-dimethylphenyl) semicarbazone.

Figure 1 summarizes the key role of the host mechanisms for viral infection and propagation, as discussed above and elsewhere [54], also postulating the role of a collection of host-targeting drugs as antivirals.

## 3. Host-Targeted Antivirals against RNA Viruses

**Figure 2:**
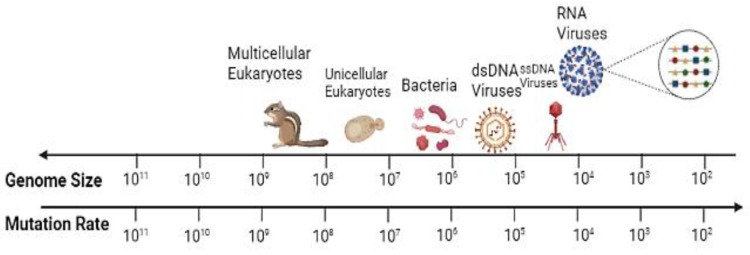
Biological mutation rate compared with genome size. RNA viruses have higher mutation rates. Adaptive mutations are highlighted by colored symbols, illustrating the genetic variation present in the RNA virus quasispecies. Data are adapted from [66].

From a phylogenetic perspective, RNA viruses evolve at a much faster rate than their hosts (Figure 2). As discussed above, this is due to the lack of proofreading of the RNA-dependent RNA polymerases (RdRp) and reverse transcriptases (RT) [64], resulting in an error frequency approximately three orders of magnitude higher than that of DNA-dependent DNA polymerases [65].

RNA viruses exist in nature as quasispecies, which can be defined as complex distributions of genomes that exhibit genetic variations that endow the mutation carriers with advantages to survive and thrive over strains that do not carry the mutation. Genetic variations emerge during non-proofed replication into host organisms, enhancing the ability of specific quasispecies to proliferate, infect more easily, evade immunity, and become resistant to treatment and/or vaccination [7]. Quasispecies that do not enjoy such an advantage are eventually eliminated. While replication fidelity is a crucial element in the survival of one quasispecies over another, additional factors, e.g., the genome architecture and speed of genome replication, may influence these evolutionary adaptations [67].

**Figure 3:**
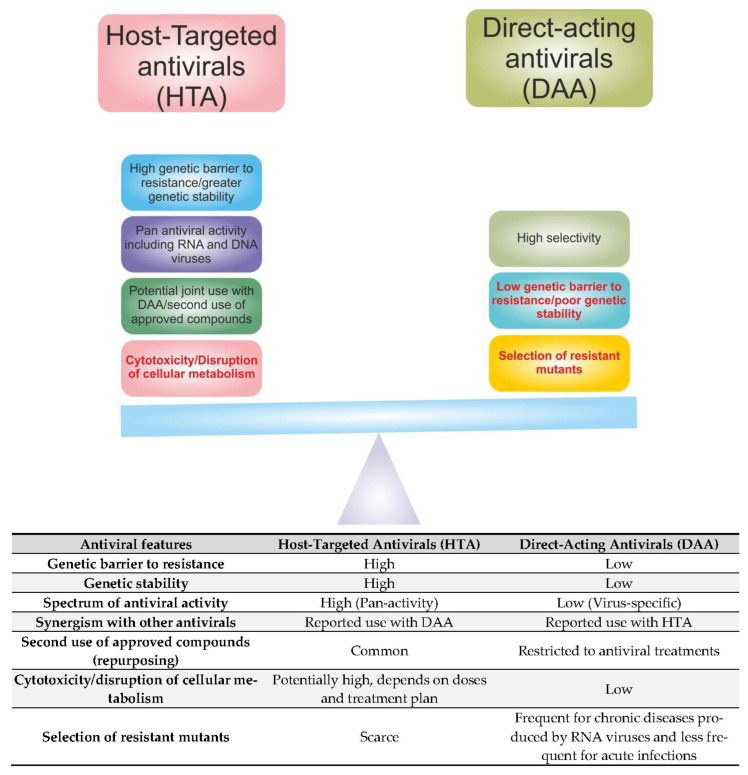
Graphic summary and summary table of the most salient features of HTAs and DAAs. Red text indicates the disadvantages of the indicated approach.

Direct-acting antivirals (DAAs) are employed to treat RNA virus infections. However, quasispecies emergence triggers the rapid appearance of resistant populations. When the RdRp inhibitor balapiravir was used to treat DENV infection, no differences were found in the plasma viral load, cytokine concentrations, and fever clearance time between the treated group and the control, indicating the resistance of the DENV quasispecies to its action [68]. On the other hand, host-targeted antivirals (HTAs) constitute a novel strategy that may overcome this issue. The main reason is that host factors are genetically more stable than viral factors, and the mutation rate of cellular genes is very low in comparison with the genes of RNA viruses [69]. Moreover, HTAs have pan-genotype and serotype antiviral activity and complementary mechanisms of action to DAAs in clinical development; thus, they could be used jointly [70]. Additionally, many HTAs are already FDA-approved drugs for the treatment of other pathologies, e.g., cardiovascular diseases, inflammatory diseases, and cancer. This means that toxicity and other adverse effects were assessed and reported beforehand [71]. However, this does not rule out the emergence of quasispecies that bypass the cellular effector targeted. This is a distinct possibility that needs to be addressed on a case-by-case basis.. Figure 3 displays a graphic representation of a scale that compares the advantages and disadvantages of HTAs with those of DAAs. In our view, the net balance of HTAs outweighs that of DAAs, hence tipping the scale toward the former.

### 3.1. Attachment and Entry Inhibitors

The endpoint of the virion entry for most RNA viruses is the release of the viral genome into the host cell cytoplasm; hence, the disruption of the initial attachment and entry of viruses can prevent the next steps of the viral replication cycle. There are a few genetic examples of this mechanism. For example, naturally occurring mutations of the chemokine receptor and HIV-1 entry co-factor CCR5 prevent infection [72,73,74]. Thereby, targeting mechanisms of entry into the host cell could be a viable antiviral strategy. A key example is HIV-1, which enters lymphocytes, macrophages, and neurons via CD4 (main receptor) and CCR5/CXCR4 (co-receptors) [75]. Maraviroc is a CCR5 antagonist that can block HIV infection [76]. However, HIV-1 can also use CXCR4 as a co-receptor. In fact, plerixafor (AMD3100), which is currently used for stem cell mobilization in MM patients undergoing autologous treatment [77,78], was originally conceived as a form of anti-HIV treatment [79]. Some examples are already available in the literature. Chebulagic acid and punicalagin compounds inhibit viral glycoprotein interactions with cell surface glycosaminoglycans in the 10–100 µM range [80], which are employed by many viruses as primary entry factors, including DENV and HCV. Similarly, λ- and ι-carrageenans act as heparan sulfate (HS)-mimetic compounds and are potent (IC_50_ = 0.14 to 4.1 μg/mL) inhibitors of DENV-2 and DENV-3 in monkey and human cells [81]. Likewise, other sulfated polysaccharides display antiviral activity against HIV and HCV [82]. Bromobenzaldehyde N-(2,6-dimethylphenyl) semicarbazone (EGA) inhibits influenza A entry into host cells by blocking the acidification of endosomes [83].

Importantly, SARS-CoV-2 infection of the host cell is enabled by the interaction of the Receptor-Binding Domain (RBD) of the SARS-CoV-2 spike (S) protein with the cellular ACE2 receptor. This makes ACE2 a potential antiviral target. Chloroquine (CQ), a 4-aminoquinoline base that causes an increase in the lysosomal pH, was suggested as a potential antiviral agent for SARS-CoV at a relatively low dose (IC_50_ ≈ 1 µM) [84], although its real-life efficacy is questionable at best [85]. Its potential mechanism of action is based on the fact that CQ inhibits the acidification and maturation of the endosome, thereby blocking the pathway in the intermediate stages of endocytosis and preventing the further transport of virus particles to the final release site [86]. CQ could also impair the terminal glycosylation of the ACE2 receptor used by SARS-CoV-2 [87], reducing the affinity of the S protein for ACE2. Additional HTAs targeting this mechanism were screened, including NAAE (N-(2-amino-ethyl)-1 aziridine-ethanamine). NAEE displayed a dose-dependent inhibition of the ACE2 catalytic activity, blocking cell fusion with the S protein. MLN-4760 is a potent (IC_50_ = 0.44 nM) inhibitor for ACE2 that forms a stable complex with ACE2 [88]. However, there is no well-established consensus on the feasibility of ACE2 receptor inhibition due to its important physiological role, including its protective effect on lung injury in acute respiratory diseases [89].

Cathepsin L and TMPRSS are suitable targets to block the entry of some viruses, e.g., SARS-CoV-2 [90,91], into cells. Teicoplanin, a glycopeptide antibiotic, inhibits cathepsin L, blocking the entry of SARS-CoV pseudotyped viruses and SARS-CoV-2 in vitro with a very low IC_50_, approximately 300 nM [92]. Likewise, oxocarbazate suppresses cathepsin L and inhibits the entry of SARS-CoV and Ebola virus with an IC_50_ also in the 300 nM range [93].

### 3.2. ER α-Glucosidase Inhibitors

Endoplasmic reticulum α-glucosidase inhibitors efficiently disrupt the morphogenesis and assembly of multiple enveloped viruses. The main reason is that these enzymes are essential for the processing, proper folding, and function of many viral glycoproteins that are part of the viral capsid.

The naturally occurring iminosugar castanospermine inhibits all serotypes of DENV in vitro with various IC_50_ values [94], also decreasing mortality in an in vivo model [95]; this compound also inhibits cell adhesion and cell-to-cell spread, glycoprotein processing, and the replication of HIV [96]. Celgosivir, a pro-drug stemming from castanospermine, conferred full protection to mice infected with DENV (IC_50_ = 5 µM) [94]. A pre-clinical trial in mice showed that celgosivir enhanced survival, reduced viremia, and promoted a vigorous immune response [97]. Celgosivir also displayed antiviral properties against HCV [98].

More recently, some iminosugar derivatives were shown to display activity against DENV and influenza virus. UV-4B protected mice from a DENV-2 lethal challenge, with IC_50_ = 17 µM [99]. It also promoted survival in a lethal influenza virus mouse model [100].

To address the advantages or disadvantages of HTA therapy, Plummer and coworkers conducted the first evolutionary study to investigate the evolution of DENV-2 under selective pressure by UV-4B [15]. This study revealed that DENV does not acquire mutations that increase fitness during in vivo replication in the presence of the compound, indicating that host factors display a high genetic barrier during the treatment of DENV infections.

### 3.3. Lipid Synthesis Inhibitors

During infection of enveloped viruses, the host’s lipid synthesis pathways are co-opted in several steps of the virus replicative cycle. Statins inhibit the mevalonate pathway, blocking the enzyme HMG-CoA reductase, which plays a central role in the production of cholesterol and is employed regularly in patients with hypercholesterolemia [101]. Previous studies performed in our laboratory showed that lovastatin inhibited DENV particle assembly (IC_50_ = 10–50 µM) in a cell culture via the inhibition of the prenylation of Rho and Rab GTPases, which are important in DENV morphogenesis [102]. A delay in infection and an increase in survival rates was observed in AG129 mice infected with DENV-2; these results were the basis of a randomized, double-blind, placebo-controlled trial in adults with dengue that demonstrated the potential use of lovastatin in anti-DENV therapy [103]. Likewise, lovastatin was effective in clinical trials to control HIV-1 replication in chronically infected individuals who were not receiving antiretroviral medication [104].

The antiviral activity of statins on the HCV subgenomic replicon showed that mevastatin and simvastatin exhibited the strongest anti-HCV activity, whereas fluvastatin and lovastatin had moderate inhibitory effects. Moreover, the combination of statins with several selective HCV inhibitors resulted in a pronounced antiviral effect in a cell culture that prevented or delayed the emergence of drug-resistant variants [105]. Simvastatin exhibited antiviral effects against influenza A virus by modulating common cellular pathways, especially during endocytosis and lysosomal activity, affecting the autophagosome function [106]. Fenretinide (4-HPR) is a synthetic retinoid that alters ceramide homeostasis. 4-HPR displayed in vitro antiviral activity against some flaviviruses, including DENV, West Nile, Modoc, and HCV viruses, and in vivo activity against DENV, with the IC_50_ in the 1–50 µM range [107].

### 3.4. Antagonists of Cytoskeletal Polymerization 

Several studies have shown that actin filaments and microtubules interact with viral particles at several steps of the replicative cycle. Pentagalloyl glucose decreased the intracellular levels of cofilin [108], blocking the reorganization of the actin cytoskeleton and influenza virus assembly and budding with an IC_50_ around 10 µM [109]. Importantly, protruding actin-based structures such as Cdc42-dependent filopodia [110] seem particularly important in viral dissemination. In this regard, ZCL278 had potent antiviral effects against junin virus (JUNV), vesicular stomatitis virus (VSV), lymphocytic choriomeningitis (LCM) virus, and dengue virus [111], by inhibiting the Cdc42 function in host cells (IC_50_ ≈ 50 µM) [112]. During DENV-2 infection, nocodazole and cytochalasin D significantly inhibited the production of infectious particles both before and after infection [54]. Similar results were reported by Wang et al. [113], who showed that cytochalasin D and jasplakinolide reduced virion ingress during treatment in early events of infection and reported an accumulation of the E viral protein later in infection. Other researchers have emphasized the importance of the interactions between the cytoskeleton and flavivirus replication cycles, suggesting the cytoskeleton as a viable target for the design of new antiviral therapies [50].

### 3.5. Approved Small Antitumor Molecules with Antiviral Effects

Mutations to multiple protein kinases underlie the initiation or development of various forms of cancer [114]. Interestingly, some of these kinases are also important mediators of viral infection; therefore, kinase antagonists (e.g., small organic molecules) developed to treat cancer could be repurposed as a therapeutic alternative to treat infections caused by RNA viruses. Some protein kinase inhibitors have antiviral activity against one or more DENV serotypes. For example, selumetinib (AZD6244) is a MEK/ERK inhibitor that displays antiviral activity against DENV-2, DENV-3, and St. Louis encephalitis virus (SLEV) by altering the virion morphogenesis in live mice (dose 100 mg/kg/day against 10^5^ CFU) [115]. Sunitinib and erlotinib interfere with the intracellular trafficking of HCV-infective particles by inhibiting AP2-associated protein kinase 1 (AAK1) and cyclin G-associated kinase (GAK). Furthermore, they produce a decrease in DENV and EBOV infection, demonstrating that these molecules could be useful as broad-spectrum antivirals [116].

Small molecules such as AZD0530 and dasatinib target the Tyr kinase Fyn with very high affinity (IC_50_ ≈ 1 nM). This kinase is involved in DENV-2 replication, thereby possessing potential anti-DENV activity [117]. Dasatinib also inhibits the activity of other Src-like kinases, exhibiting a potent inhibitory effect on dengue virus (serotypes 1–4) by preventing the assembly of dengue virions within the virus-induced membranous replication complex. Indeed, dasatinib efficiently (IC_50_ ≈ 1–5 µM) blocked DENV infection in vitro [118]. Furthermore, dasatinib prevents permeability alterations during DENV infection [119], likely by targeting the RhoA/ROCK axis [120]. ABL kinases regulate several cellular pathways, including cell migration, adhesion, and actin reorganization [121]. A prepandemic study identified imatinib as an antagonist (IC_50_ ≈ 10–50 µM) of both SARS-CoV and MERS-CoV replication in vitro [122]. Imatinib inhibited the early stages of the coronavirus life cycle by blocking viral fusion with the endosomal membrane, thus inhibiting subsequent viral genome replication. In Ebola infection, imatinib prevents the phosphorylation of the viral VP40 protein, which is necessary for virion egress from the host cell [123].

More recently, GNF-2 displayed a potent and specific effect (IC_50_ similar to that of imatinib) against DENV by inhibiting the kinase activity of Abl by allosterically binding to the myristoyl-binding pocket of Abl. However, it also displayed an Abl-independent mechanism, targeting the interaction with the viral E protein in the prefusion state [124]. Finally, ruxolitinib, an inhibitor of Janus Kinases 1/2 and an FDA-approved drug for the treatment of proliferative neoplastic myelofibrosis, reduces HIV-1 replication in human macrophages at a very low IC_50_ (0.1–0.4 µM) [125]. Together, these data provide evidence of the broad-spectrum activity of FDA-approved antitumor drugs.

### 3.6. Plant-Derived Natural Compounds

Plant-derived natural products are an important form of antiviral treatment, particularly in developing countries due to the ancient social roots of traditional medicine [126]. Many potential antivirals derived from natural products were described, of which we discuss only the best characterized. Importantly, some of these can act as direct antivirals. For example, curcumin (see below) can inhibit the DENV NS2B/NS3 protease system [127]. However, they can also target host-dependent mechanisms. This section focuses on the latter. 

Immunosuppressive drugs such as mycophenolic acid (IMP dehydrogenase inhibitor) exhibit anti-DENV activity, preventing the synthesis and accumulation of viral RNA [128]. Cyclosporine, a cyclophilin protein inhibitor and immunosuppressant agent used conventionally to prevent graft rejection, displayed antiviral activity against DENV, HCV, and HIV at the step of RNA synthesis [129,130]. FGI-106, a low-molecular-weight inhibitor, has shown broad-spectrum antiviral activity against Ebola virus both in vitro and in vivo. Although its mechanism of action has yet to be elucidated, this compound was shown to also be effective against DENV, HIV, and HCV infections [131].

Carotenoid pigments have antitumor and antiviral effects due to the activation of caspases that inhibit metalloproteinases. Treating HCV- and HBV-infected mononuclear cells with these pigments decreased the activity of viral polymerases, thereby inhibiting viral replication.

Curcumin has effects on several cellular processes and/or structures, e.g., the cytoskeleton and the ubiquitin–proteasome system [132]. It also induces apoptosis [133]. All these effects interfere with dengue virus infection [134]. On the other hand, ferruginol derivatives are active against DENV-2 in post-infection stages, resulting in a dramatic reduction in the viral plaque size [18].

A summary of host-targeted antiviral with a non-comprehensive list of compounds appears in Table 1.

## 4. Ferruginol Analogs as Potential Host-Targeted Antiviral

**Figure 4:**
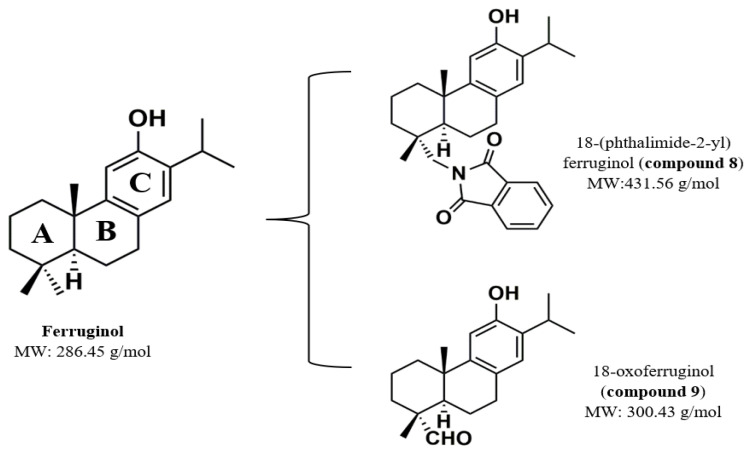
Chemical structure of ferruginol and two of its analogs, 18-(phthalimide-2-yl) ferruginol (compound 8) and 18-oxoferruginol (compound 9). MW: molecular weight.

Ferruginol is a diterpenoid phenol isolated from plants of the *Podocarpaceae*, *Cupressaceae*, *Lamiaceae*, and *Verbenaceae* families. Ferruginol has a wide spectrum of biological activities, such as antibacterial, antifungal, antimicrobial, acaricide, cardioactive, antioxidant, anti-Leishmania, antiplasmodium, nematicide, anti-ulcerous, and cytotoxic activities on tumor cells. Previously, we reported that two ferruginol analogs, 18-(phthalimide-2-yl) ferruginol (compound 8) and 18-oxoferruginol (compound 9, shown in Figure 4), reduced the in vitro infection of human herpesvirus type 1 and 2 and dengue serotype 2 when added in post-infection stages. In addition, compound 8 significantly reduced the size of the viral plaques when DENV-2-infected cells were treated [18].

18-(phthalimide-2-yl) ferruginol displays a phthalimide group in ring A of the ferruginol backbone, specifically in carbon 18, as indicated in Figure 4. Phthalimide is an imide derived from phthalic acid with two carbonyl groups joined to a secondary amine. These compounds are hydrophobic, endowing the molecule with the ability to traverse biological membranes in vivo [136]. In addition, these molecules and some of their derivatives are endowed with antibacterial, antifungal, analgesic, antitumor, anxiolytic, hypolipemic, and analgesic activities and have low in vivo toxicity [137].

Our recent data suggest that compound 8 is endowed with potential broad-spectrum antiviral activity. It does inhibit in vitro infection by Zika virus in Vero [19], PC3, and HeLa cells at concentrations below 10 μM. Likewise, this molecule has antiviral activity in Vero cells infected with CHIKV (Alphavirus genus) [138]. Further unpublished studies related to the antiviral mechanism of action of this molecule strongly suggest that 18-(phthalimide-2-yl)-ferruginol has an HTA-related mechanism of action by disrupting the DENV-2 polyprotein translation via the alteration of actin remodeling and other related cellular and viral processes involved in the replicative complex formation. Additionally, in vitro and in silico evidence indicates that this compound has few cytotoxic and potentially reversible effects on host cells.

## 5. Integration of Bioinformatics with the Search for Host-Targeted Antivirals 

As discussed above, new therapeutic strategies for RNA viruses could be based on targeting host proteins and processes that disturb the virus–cell interactomes that emerge during virus colonization of the host cell. This view integrates the fact that viruses can manipulate and modify cellular protein networks essential for the viral cycle [139,140]. Databases such as VirusMINT (https://bio.tools/virusmint accessed on 25 November 2022) and VirHostNet (https://virhostnet.prabi.fr/ accessed on 25 November 2022) and drug-target databases such as DrugBank (https://go.drugbank.com/ accessed on 25 November 2022) are powerful tools to obtain preliminary information regarding the interactions between virus and host proteins.

**Figure 5:**
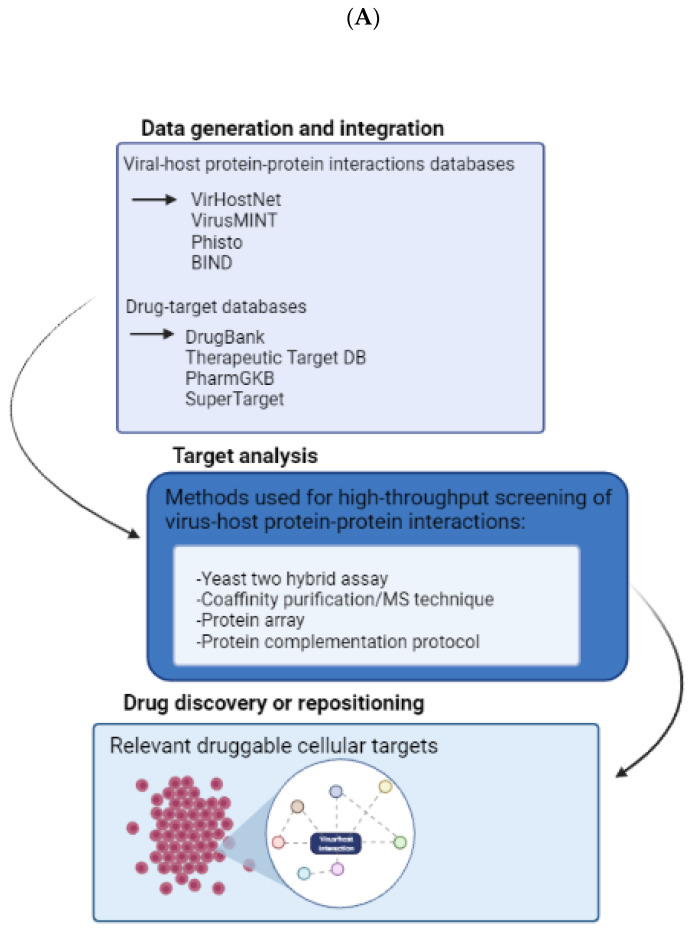

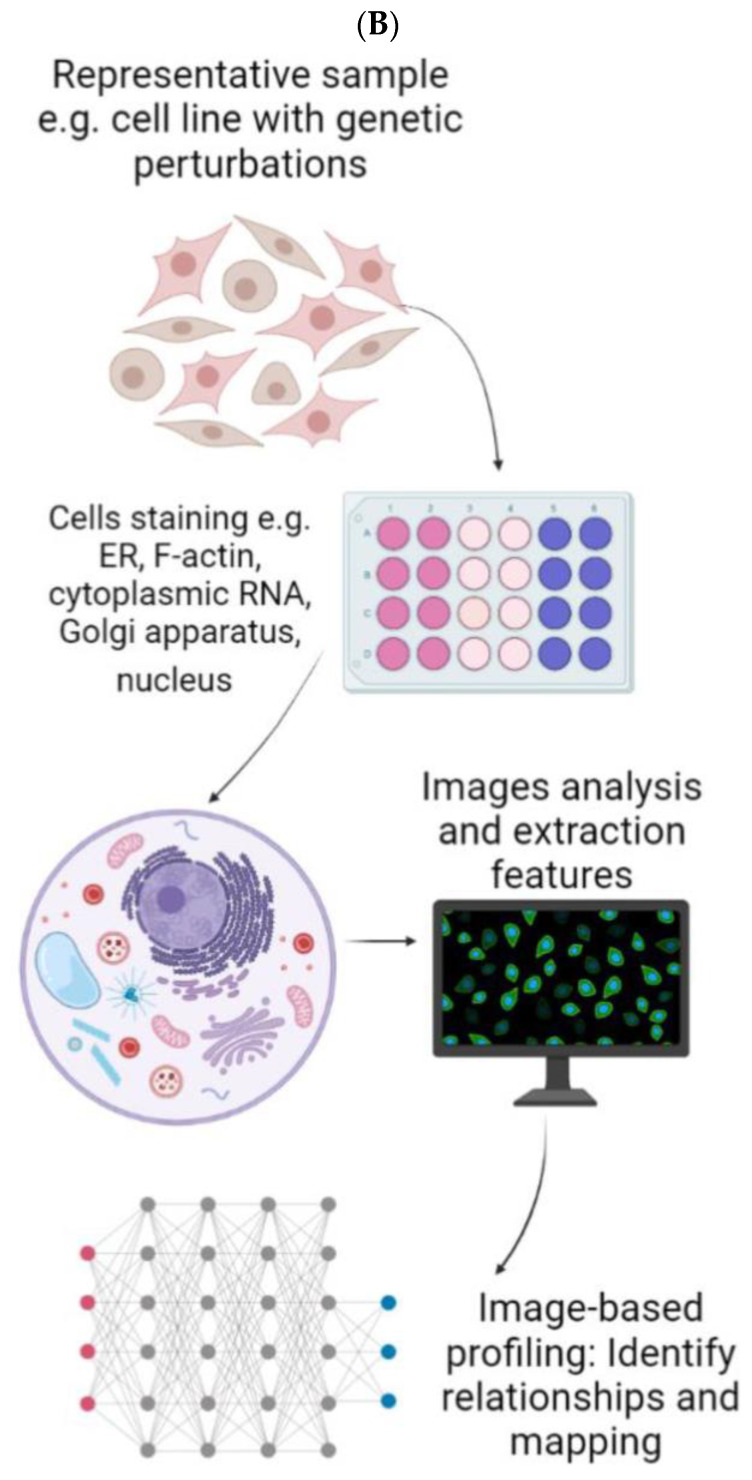
Potential pipeline for the discovery of host-targeted antivirals. (**A**) Workflow to search for cellular targets involved in virus–cell interactions. Databases provide initial identification of possible targets in the interactions between viruses and cells that need to be confirmed by experimental techniques. The next step includes repositioning existing drugs or discovering new inhibitors that may act as host-targeted antivirals. (**B**) Image-based profiling overview from biological samples.

Experimentally, the high-throughput screening of virus-host protein–protein interaction methods, such as yeast two-hybrid assays, co-affinity purification/MS techniques, protein arrays, and protein complementation protocols such as mass spectrometry, offers great benefits for expanding virus–cell interactome knowledge and provides the opportunity to discover new targets in humans [141]. Another promising technology for novel drug discovery is image-based profiling, which includes computational equipment, such as deep learning and single-cell methods; in short, these tools collect relevant biological information present in an image, reducing it to a multidimensional profile (see Figure 5B) [142]. Altogether, the focus of this outlook must be the host cell targets improving the repurposing of drugs or finding new ones, because actual treatments remain limited, inefficient, and incapable of challenging drug resistance. The workflow proposed for such a search strategy is depicted in Figure 5A.

## 6. Conclusions and Perspectives

The goal of an antiviral drug is to avoid or resolve the infection to prevent the development of severe disease or the death of the host. This kind of therapy must be effective, ensuring complete resolution of the infection. Likewise, understanding the mechanisms by which molecules exert their antiviral action not only on the virus but also on the host cell allows an estimation of the risk–benefit balance.

Moreover, there is overwhelming proof of the impact of viral escape mutants on global human health. The COVID-19 pandemic is a relevant example of the appearance of viral strains resistant to vaccination that can become massively contagious.

All these lessons show that HTA therapy is a valid alternative for the new millennium in research, not only against viruses but also in general for microorganisms that evolve faster than their hosts. It is true that HTA therapy has many potential adverse effects, and many clinical trials are ongoing to ascertain this aspect among others. Doubts that remain in this research field are more numerous than the data provided thus far but generate new hope in the field of pharmacology to design more rational drugs.

## Figures and Tables

**Table 1:** Host-targeted antivirals against some RNA viruses.

Category	Compound	Cellular Target	Virus	References
Entry and attachment inhibitors	Chebulagic acid and punicalagin	Cellular surface glycosaminoglycans	DENV, HCV	[80]
	Carrageenans	HS-imitative compounds	DENV, HIV, HCV	[81]
	Teicoplanin	Cathepsin L	SARS-CoV-2	[92]
	Oxocarbazate		SARS-CoV, EBOV	[93]
Endosomal function inhibitors	EGA	Endosome acidification	Influenza A	[83]
	Chloroquine		SARS-CoV-2, SARS-CoV	[84,86,87]
α-glucosidase inhibitors	Castanospermine	ER glucosidases/ disruption of glycoprotein processing	DENV, HIV	[94,95,96]
	Celgosivir		DENV, HCV	[99,100]
	Iminosugar derivatives		DENV, Influenza	[15,100]
Lipid synthesis inhibitors	Lovastatin	Prenylation of Rho and Rab GTPases	DENV, HCV, HIV	[102,103,104]
	Mevastatin	HMG-CoA reductase	HCV, Influenza A	[71,106]
	Simvastatin			
	Fenretinide	Ceramide homeostasis	DENV, West Nile, Modoc, HCV	[107]
Agents acting on cytoskeleton	Pentagalloyl glucose	Cofilin	Influenza	[108,109]
	ZCL278	Cdc42	DENV, JUNV, VSV, LCM	[111]
	Cytochalasin D	Actin filaments	DENV	[54,113]
	Jasplakinolide			
	Nocodazol	Microtubules		
Antitumor molecules	Selumetinib (AZD6244)	MEK/ERK	DENV, SLEV	[115]
	Sunitinib and erlotinib	AAK1/GAK	HCV, DENV, EBOV	[116]
	AZD0530 and dasatinib	Fyn/Src kinases	DENV, Modoc	[117,118]
	Imatinib	c-ABL	DENV, SARS-CoV, MERS-CoV, EBOV	[122,123]
	GNF-2		DENV	[124]
	Ruxolitinib	JAK ½	HIV	[125]
Plant-derived natural compounds	Mycophenolic acid	IMP dehydrogenase	DENV	[128]
	Cyclosporine	Cyclophilin protein	DENV, HCV, HIV	[129,130]
	FGI-106	Unknown	DENV, EBOV, HIV, HCV	[131]
	Carotenoid pigments	Metalloproteinases	HCV	[135]
	Curcumin	Cytoskeleton, ubiquitin–proteasome-proteasome system, apoptosis	DENV	[134]
	18-(phthalimide-2-yl) ferruginol	Actin remodeling, polyprotein translation, replicative complexes	DENV, ZIKV, CHIKV	[18]

HS: heparan sulfate; ER: endoplasmic reticulum; HMG-CoA: 3-hydroxy-3-methylglutaryl coenzyme A reductase; IMP dehydrogenase: Inosine-5’-monophosphate dehydrogenase; EGA: bromobenzaldehyde N-(2,6-dimethylphenyl) semicarbazone; MEK/ERK: mitogen-activated protein kinase kinase/extracellular signal-regulated kinase; AAK1/GAK: AP2-associated protein kinase 1/cyclin G-associated kinase; Fyn/Src: proto-oncogene tyrosine-protein kinase; JAK: Janus kinase.
